# 

**Published:** 1871-01

**Authors:** 


					﻿Vol. i. 'V.. '	January. 1S71.	No. i.
• • < //
THE GEORGIA '
Medical Companion,
.1 MONTHL T ,l/)UZS777x\
DEVOTED TO THE
SCIENCE OF MEDICINE AND SURGERY.
EDITED BY'
T. S. POWELL, M. D.,
W. T. GOLDSMITH, M. D.
QUIC^UID PRPECIPIES ESTO BREVIS." 7^^
CONTENTS.
PART I.—Original Communications and Special Selections.
PART IL—Abridged Extracts’ and Gleanings from Our Exchanges.
PART III;—Editorials, Reviews, Items, and NEWS.^rrgg^
ATLANTA. GA.
Franklin Steam Printing House.
J. J. TOON, Proprietor.
Terms, ...	-	■ S2 a Year in Advance.
				

